# Successful pregnancy outcome via in-vitro fertilization and laparoscopic resection of non-communicating rudimentary horn pregnancy containing early pregnancy: a case report

**DOI:** 10.1186/s12884-024-06289-2

**Published:** 2024-02-07

**Authors:** So Yeon Shin, Hwang Kwon, Hyun Chul Kim, Min Jung Baek, Ji Eun Shin

**Affiliations:** 1 Department of Obstetrics and Gynecology, CHA Fertility Center Bundang, 59, Yatap-Ro, Bundang-Gu, Seongnam-Si, Gyeonggi-Do 13496 Korea; 2 Department of Obstetrics and Gynecology, CHA Women’s Hospital Bundang, Seongnam-si, Gyeonggi-Do 13496 Korea

**Keywords:** Mullerian anomaly, Rudimentary horn pregnancy, Hysterosalpingography, Assisted-reproductive technology, In-vitro fertilization

## Abstract

**Background:**

Non-communicating rudimentary horn pregnancy (NCRHP) lead to life-threatening condition for both mother and fetus. Early diagnosis of NCRHP and laparoscopic resection is important to prevent catastrophic conditions. However, delayed diagnosis until the second or third trimester makes it difficult to accurately diagnose between NCRHP and bicornuate uterine pregnancy, as both conditions present uterine rupture and massive hemoperitoneum. Furthermore, these rare cases are challenging in pregnancy trials and associated with adverse outcomes in subsequent pregnancies.

**Case presentation:**

A 31-year-old gravida 1 para 0 Korean woman visited our infertility center with a confirmed positive urine pregnancy test after timed intercourse. Before she was scheduled to have timed intercourse, a unicornuate uterus with a non-communicating right uterine horn was suspected based on an ultrasound scan and hysterosalpingography during the initial infertility workup. A gestational sac was observed in the right non-communicating rudimentary horn at 5 weeks of gestation. Serum beta-human chorionic gonadotropin (b-hCG) level was 2052.0mIU/mL. An elective laparoscopic resection of the right rudimentary horn containing a gestational sac, along with ipsilateral salpingectomy, was performed with no adverse event. After 3-month of recovery period and three cycles of conceptional trials involving timed intercourse and intrauterine insemination, in-vitro fertilization (IVF) was performed using the antagonist protocol, and successful pregnancy was confirmed. The patient had been hospitalized from 21 + 6 weeks to 35 + 6 weeks of gestation, underwent cerclage placement and tocolytics with corticosteroid treatment. She delivered an early-term male baby by cesarean section.

**Conclusion:**

In this rare case, the successful pregnancy achieved through IVF following the appropriate management of NCRHP under laparoscopy underscores the critical importance of early diagnosis and intervention in cases of NCRHP. Timely identification and management of NCRHP are vital to prevent the occurrence of catastrophic conditions and to enhance the prognosis of a successful pregnancy through assisted reproductive technology (ART). Therefore, a high index of suspicion for NCRHP is important and employs a range of diagnostic modalities.

## Background

Non-communicating rudimentary horn pregnancy (NCRHP) is an extremely rare condition, with a reported incidence of 1:76,000 to 1:150,000 with 75 to 83% of pregnancies in non-communicating uterine horn [1]. The possible mechanism for non-communicating horn pregnancy is explained as the theory of transperitoneal migration of spermatozoa or fertilized ovum [2]. In most of the reports regarding NCRHP, the patients were in life-threatening conditions for both the mother and the fetus because of intrauterine growth restriction, uterine rupture, and hemoperitoneum [3, 4]. It is indicated that only 8% of rudimentary horn pregnancies are diagnosed before the critical symptom [5].

Delayed diagnosis of NCRHP makes accurate diagnosis and treatment of NCRHP difficult in case of uterine rupture and massive hemoperitoneum. Also, even with proper management, successful pregnancy after the treatment of NCRHP is very challenging and rare [1, 6]. The advent of assisted reproductive technology (ART) might contribute to a lot of pregnancy success.

The incidence rate of NCRHP is very low, so these rare cases are reported as case reports in the literature. However, to our best knowledge, there has been no report of pregnancy trials using assisted reproductive technology after the treatment of NCRHP. Here, we introduced the first case of successful pregnancy through in vitro fertilization (IVF) after proper management of NCRHP under laparoscopy.

## Case presentation

**Fig. 1:**
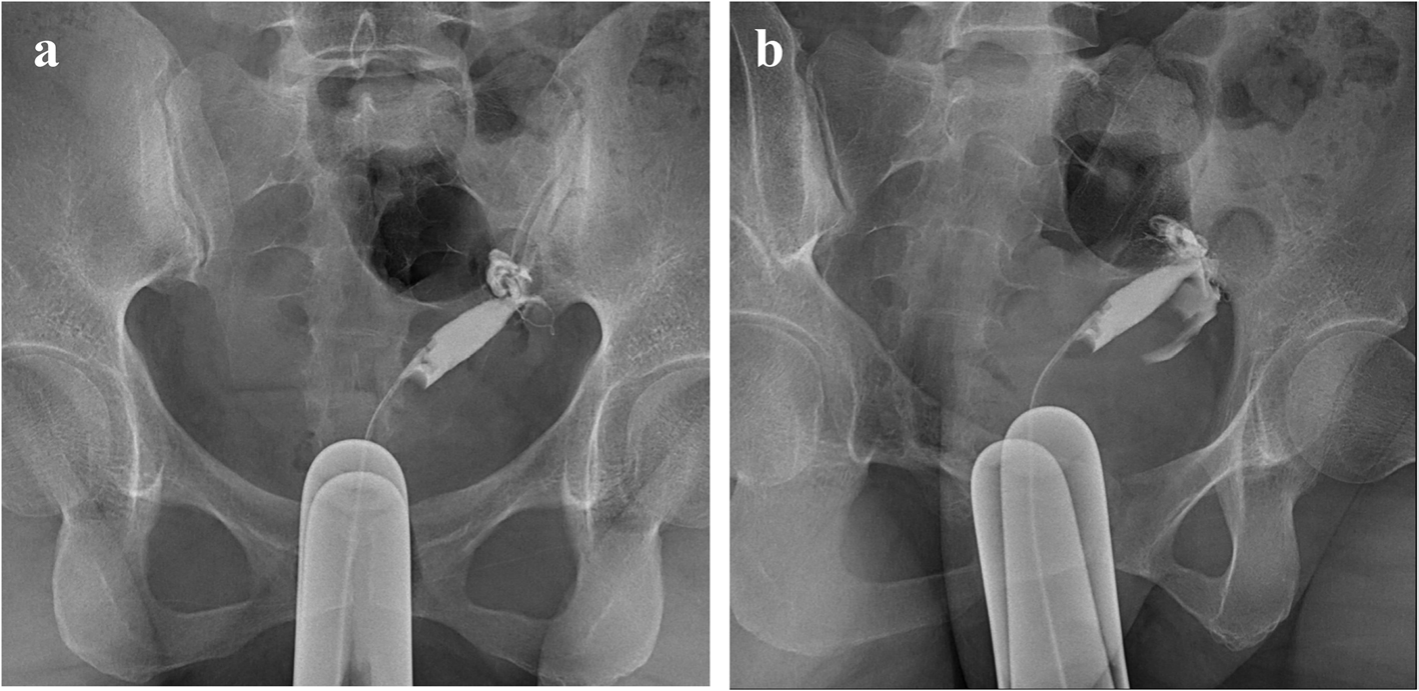
Hysterosalpingography showed a unicornuate uterus and left single salpinx (**a** and **b**). The contrast media showed a single uterine cavity suggesting the patient has a unicornuate uterus with non-communicating rudimentary horn

**Fig. 2:**
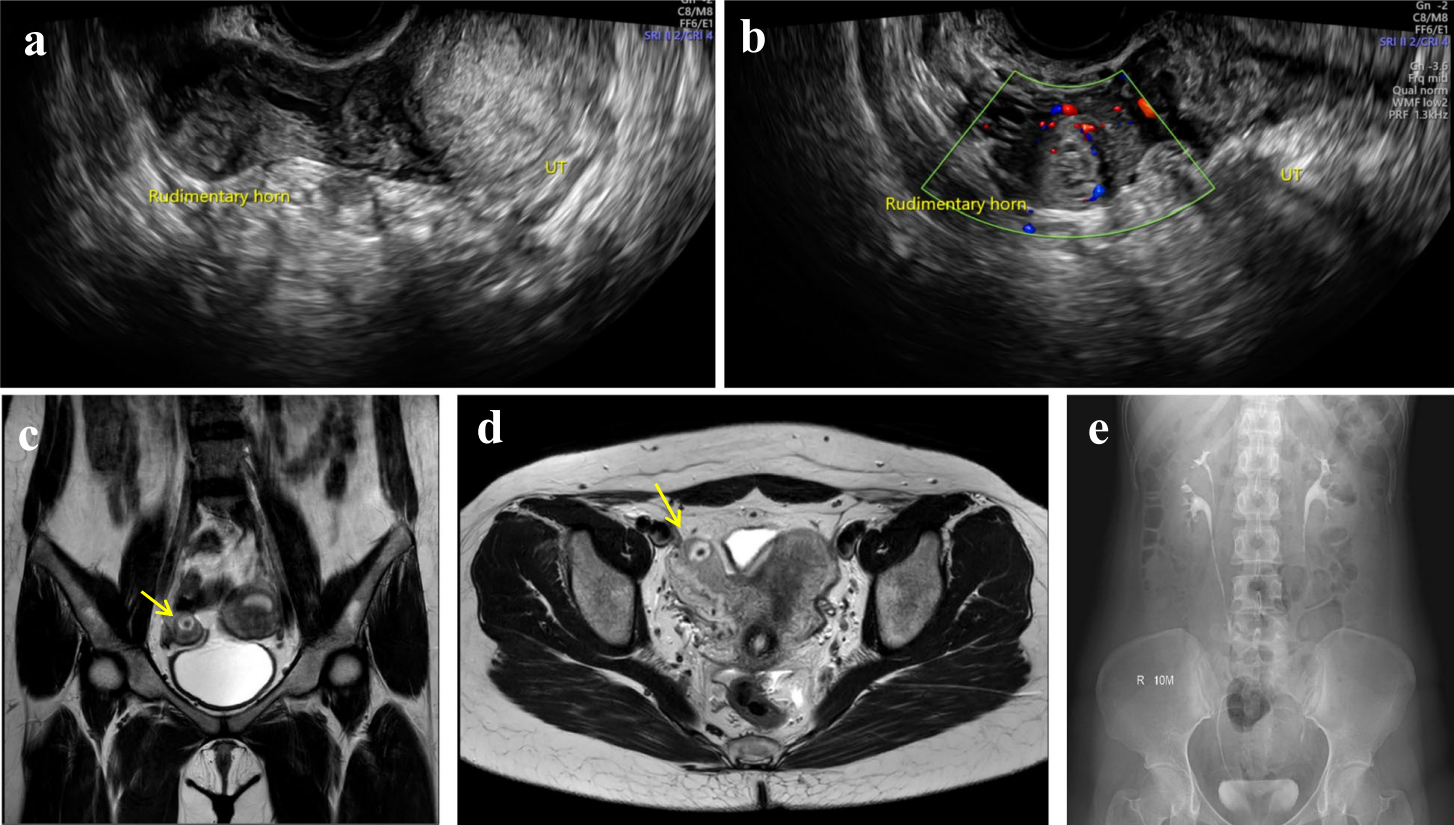
Transvaginal ultrasound (TVS) was examined, which diagnosed the left unicornuate uterus with the right rudimentary horn (**a**). Increased vascularity and double decidual sign suggesting right rudimentary horn pregnancy (**b**). T2-weighted Magnetic resonance imaging (MRI) of the pelvis showed about 8 mm-sized relatively thick-walled cystic lesions at the cornus of the right rudimentary horn or right salpinx proximal portion, suggesting RHP (**c** and **d**). Intravenous pyelogram revealed right duplex kidney (**e**)

A 31-year-old woman visited our fertility center after confirming her pregnancy with a urine test. She was previously diagnosed with secondary infertility, having not conceived since a missed abortion a year prior. An Initial infertility workup, which included an ultrasound, hysterosalpingography (Fig. 1), and prenatal test with hormonal lab tests was conducted. She was diagnosed with Müllerian duct anomaly, a unicornuate uterus with a non-communicating right rudimentary horn, classified as U4aC0V0 according to the ESHRE/ESGE classification system. Given that the patient had a patent left salpinx, a normal-sized left-sided unicornuate uterine cavity with normal endometrium, and a regular 30-day interval menstrual cycle, the doctor recommended intercourses on cycle days 19 and 21 to maximize fecundity. Three weeks later, she returned to our clinic to confirm a normal intrauterine pregnancy after her positive urine pregnancy test. Based on her estimated ovulation date, she was considered to be at 5 weeks and 1 day of gestation. A blood test revealed a beta-human chorionic gonadotropin (b-hCG) concentration of 2052.0mIU/mL. A transvaginal sonography (TVS) exam demonstrated a hyperechoic gestational sac-like lesion with increased vascularity, as indicated by the color Doppler. The lesion was surrounded by the myometrial wall of the right-sided rudimentary horn (Figs. 2a and b) and was dated at 5 weeks and 6 days of gestation. The next day, at 6 weeks of gestation, the patient was admitted for magnetic resonance imaging (MRI), intravenous pyelogram (IVP), monitoring and preventing hemodynamically unstable circumstances, such as uterine rupture, and to plan for laparoscopic surgery. The MRI revealed an approximately 8 mm-sized, thick-walled cystic lesion at the cornua of the right rudimentary horn (Figs. 2c and d), suggesting a pregnancy in the right rudimentary horn, consistent with previous transvaginal ultrasound. A corpus luteal cyst was noted in the right ovary, and the thickened endometrium showed no pathologic findings in the left unicornuate uterus. IVP revealed the right duplex kidney (Fig. 2e). The serum b-hCG concentration had increased to 9269.0mIU/mL. Based on the imaging studies and serum b-hCG concentration, a diagnosis of non-communicating rudimentary horn pregnancy (NCRHP) was made. The patient and her husband were informed of the potentially life-threatening risks of continuing the pregnancy. They were advised that the safest treatment would be an elective laparoscopic resection of the rudimentary horn. After understanding the risks, the patient and her husband signed up for the informed consent of laparoscopic resection of the rudimentary horn and ipsilateral salpinx.

**Fig. 3:**
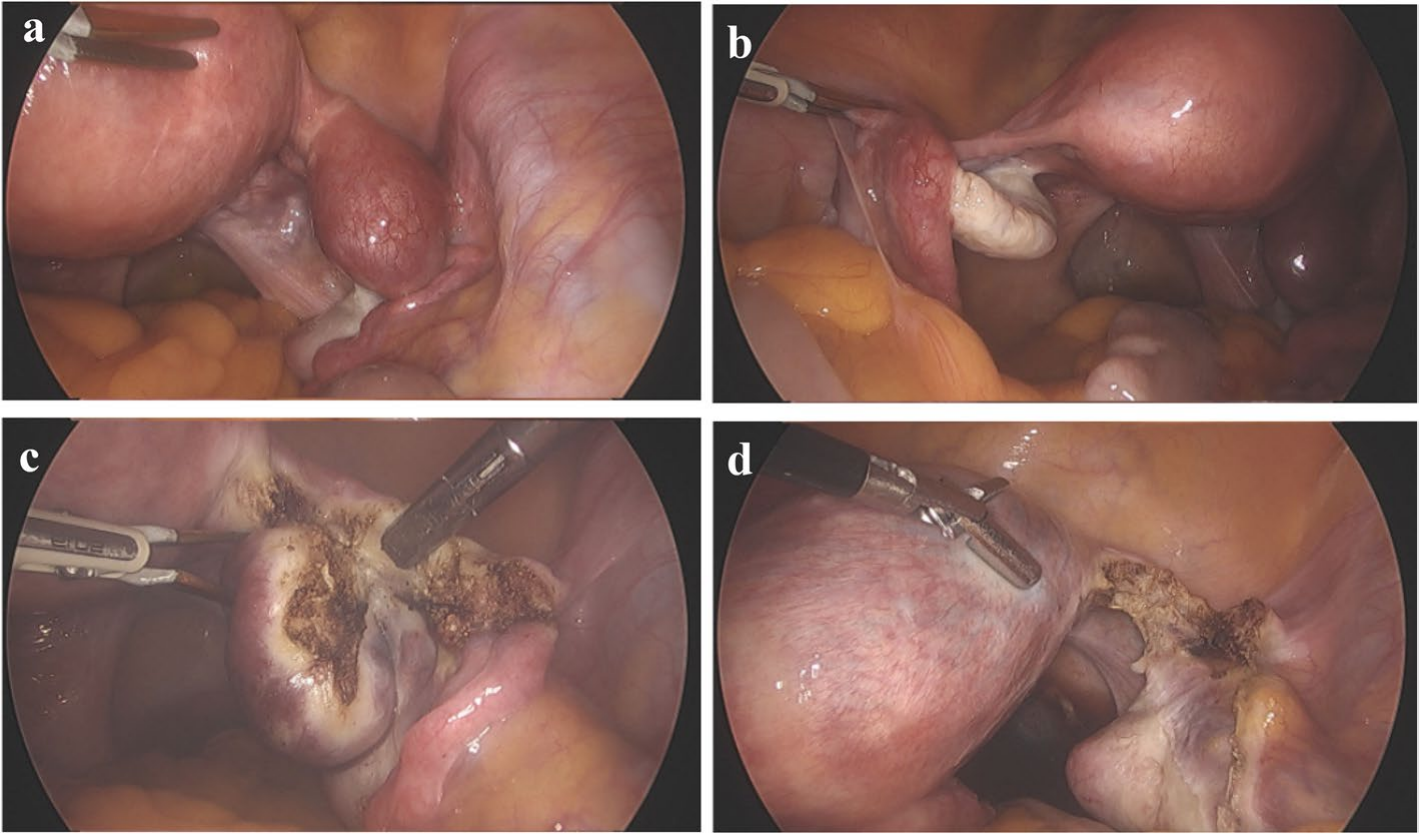
Intraoperative laparoscopic images of a non-communicating rudimentary horn, with no sign of hemoperitoneum (**a**) and contralateral left adnexa connected uterus (**b**). Laparoscopic horn excision and prophylactic salpingectomy were successfully performed (**c** and **d**)

The following day, laparoscopic resection of the rudimentary horn and ipsilateral salpinx was performed. Upon entering the abdominal cavity with one 10-mm trocar at the umbilicus, and two additional 5-mm trocars at the bilateral lower quadrants, no ascites or signs of bleeding were identified. The surface of the right rudimentary horn, which contained the gestational sac, appeared engorged with pronounced peripheral vessels. The right salpinx originated from the horn, while the left adnexa was grossly normal and connected to the dominant left unicornuate uterus (Fig. 3a and b). The right rudimentary horn and right salpinx were completely resected using a bipolar coagulator (Fig. 3c). Indigo-carmine dye was infused to confirm the connectivity between the two horns of the uterus, which was verified as non-communicated (Fig. 3d).

After the surgical treatment, the patient was discharged on postoperative day 3 without any complications. The postoperative b-hCG concentration was 1135.0mIU/mL on the day of discharge and gradually decreased to 6.820mIU/mL 18 days after resection. The histological report confirmed the presence of the product of conception inside the rudimentary horn.

**Fig. 4:**
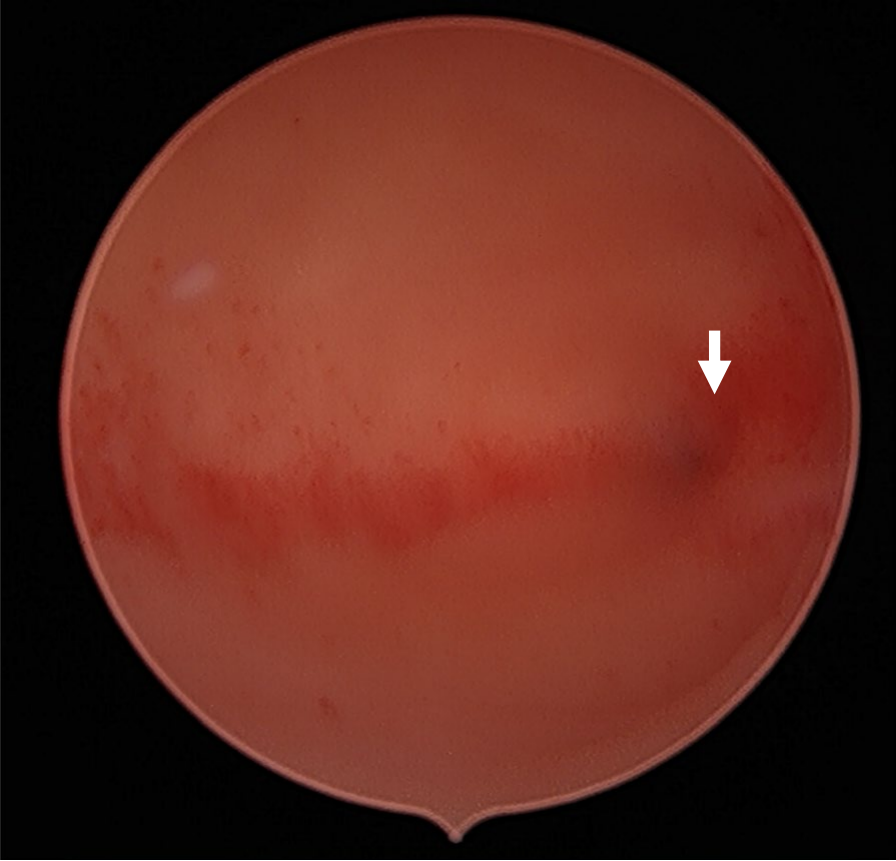
Diagnostic hysteroscopy images of unicornuate uterus with intact endometrial lining

**Fig. 5:**
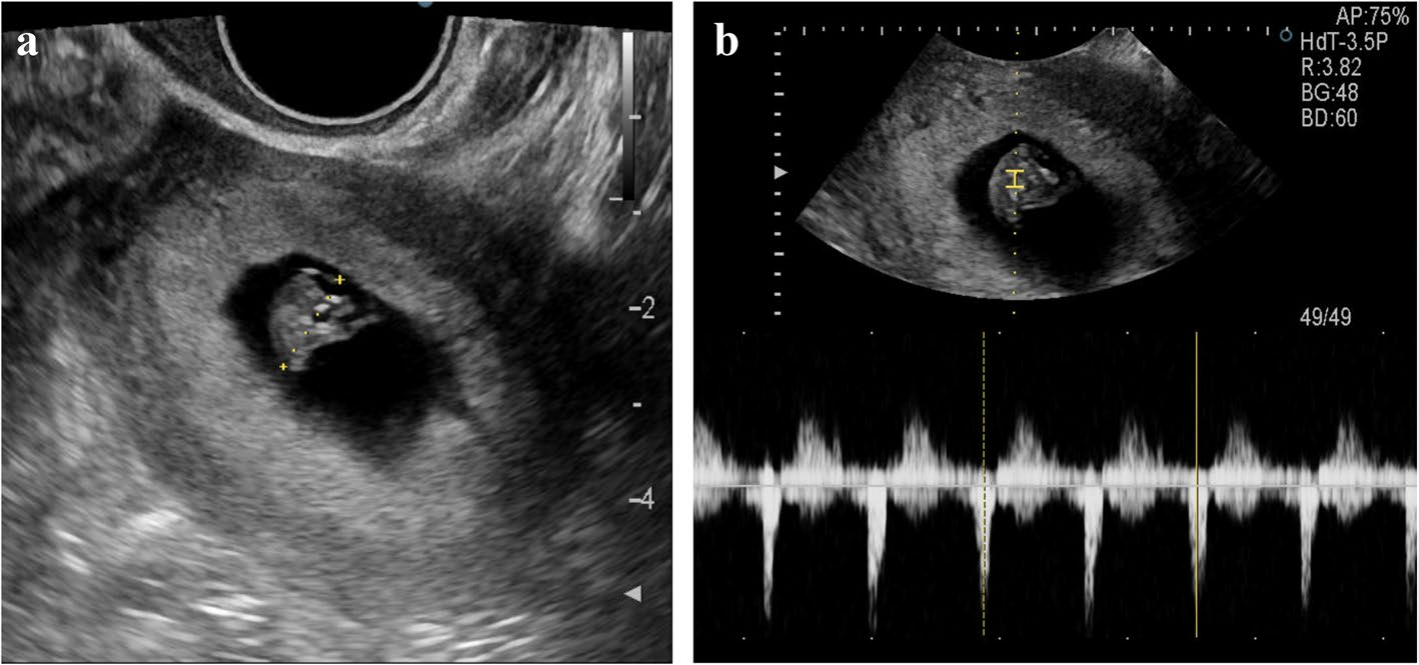
Transvaginal ultrasound images of confirmation of intrauterine pregnancy after frozen-thawed elective single embryo transfer. The Crown-Rump length (**a**) and fetal heartbeat were measured (**b**)

Three months after her recovery, she returned to our fertility center to attempt natural conception through superovulation and timed intercourse. After two unsuccessful cycles of timed intercourse and one cycle of intrauterine insemination, she opted for IVF. During her initial assessment, the serum anti-Müllerian hormone level was 3.63 ng/mL, and the follicle-stimulating hormone on day 3 was 9.02mIU/mL. Using a regular dose of gonadotropin with an antagonist protocol, we collected eleven oocytes, fertilized seven embryos, and obtained four blastocysts, three of which were good-quality embryos. Diagnostic hysteroscopy before the frozen-thawed embryo transfer cycle revealed a single external os of the cervix, a left uterine cavity with an intact endometrial lining, and a single left tubal ostium. Endometrial scratching with hysteroscopic biopsy forceps was performed (Fig. 4). The following menstrual cycle endometrium was prepared with hormone replacement (estradiol valerate 6 mg/day), and a single blastocyst was transferred. A successful pregnancy through IVF was confirmed with a first-trimester transvaginal ultrasound (Fig. 5).

At 21 weeks and 6 days of gestation, she was hospitalized due to a short cervix and signs of preterm labor. She underwent cerclage and received intravenous tocolytic therapy and corticosteroid treatment during her hospital stay. She remained hospitalized until 35 weeks and 6 days of gestation and was discharged after cerclage release. A week later, an outpatient assessment revealed her amniotic fluid index was 3.2. She was readmitted for induction. She delivered a healthy male baby weighing 2495 g with APGAR of 7 at 1 min and 9 at 5 min at 37 weeks and 0 days by cesarean section due to failure to progress.

## Discussion and conclusions

NCRHP is an extremely rare disease, predominantly reported in case reports or case series. A recent review article, encompassing 103 cases from 29 countries, found that 38 (36.9%) cases were identified in the first trimester of gestation. Most of the patients were asymptomatic and were successfully treated with laparoscopy. However, this case series did not include the details of post-treatment pregnancy outcomes [7]. To our knowledge, this study presents the first case of successful pregnancy through IVF after proper management of NCRHP suggesting the applicability of ART.

In our case, NCRHP was suspected at 5 weeks of gestation during the initial ultrasound examination. Hence, the patient received timely treatment without significant complications. Delayed diagnoses, as mentioned in the literature, often lead to more severe symptoms and outcomes during the second or third trimesters, such as abdominal pain, hemoperitoneum, and hypovolemic shock, indicating potential uterine rupture or massive blood loss requiring emergency surgical intervention [7]. These scenarios usually result in low fetal viability and increased maternal complications and mortality.

In previous studies, rudimentary horn pregnancies were often discovered incidentally during routine antenatal ultrasound screenings or emergency surgeries following the manifestation of abdominal pain [7]. Unlike those cases, our patient underwent a transvaginal ultrasound and hysterosalpingogram for an infertility workup during her initial screening. We were already aware of her unicornuate uterus with a non-communicating rudimentary horn. Thus, upon the Initial ultrasound to confirm the pregnancy, we promptly diagnosed the rudimentary horn pregnancy. This emphasized the importance of thorough preliminary screening for Müllerian abnormality in subfertility couples visiting fertility centers. It is difficult to distinguish between rudimentary horn pregnancy and bicornuate uterus unless these are recognized before pregnancy [1].

If uterine malformation is suspected, comprehensive evaluations, including ultrasound, three-dimensional ultrasound, MRI, and hysterosalpingography, can increase early and accurate diagnosis, reducing potential pregnancy complications. Our early suspicion and use of multiple diagnostic modalities allowed us to diagnose the condition during the first trimester. Therefore, clinicians should be vigilant and consider NCRHP, employing various diagnostic tools as early as possible.

Existing literature suggests that pregnancy outcomes for women with a unicornuate uterus are relatively unfavorable compared to those with a normal uterus [8]. As far as we know, this is the first case of pregnancy through IVF following treatment of NCRHP. Had the NCRHP not been identified early in the pregnancy, the risks of uterine rupture and endometrial defects would have been elevated, likely affecting subsequent pregnancy outcomes. Accordingly, we could suggest that ART was the appropriate treatment for this patient who has only one patent fallopian tube and a less expansive uterine cavity, particularly after laparoscopic rudimentary horn resection and ipsilateral salpingectomy.

Resection of the rudimentary horn and ipsilateral salpingectomy are widely accepted to prevent ectopic and tubal pregnancies. However, Isono et al. reported 18 cases identified in the third trimester, with 12 resulting in live births. The number of patients experiencing abdominal pain was significantly lower than those identified in the second trimester. Surgical resection is suitable for patients diagnosed in the first and second trimesters. Conversely, NCRHP identified in the third trimester requires careful consideration, weighing fetal viability against material risks. If a patient is in the third trimester and exhibits no specific symptoms, obstetricians might opt to continue the pregnancy with close observation and fetal surveillance, waiting until 34 weeks of gestation for fetal pulmonary maturation. However, for patients in the first and second trimesters, their risks of uterine rupture and significant bleeding outweigh the chances of viable delivery, suggesting these patients should undergo resection. Moreover, renal anomalies accompanied 29 to 44% of these patients [9]. If elective surgery is scheduled as in our case, a urinary system evaluation should be preceded to prevent intraoperative ureter injury.

Pregnancies in women with a unicornuate uterus are recognized as high-risk due to an increased likelihood of preterm births. A recent study comparing women with a unicornuate uterus to a control group matched for BMI and age found increased rates of both miscarriage and preterm delivery, with an adjusted odds (aOR) ratio of 3.04 [8]. The underlying factor for these adverse outcomes is believed to be the relatively limited size of the unicornuate uterus, leading to insufficient expansion during pregnancy. The patient in this case, who became pregnant after laparoscopic resection of NCRHP, ultimately underwent early-term delivery after being admitted for a short cervix and preterm labor. It is hypothesized that early detection and resection of NCRHP in an unruptured state contributed to the favorable pregnancy outcome. Nevertheless, if there had been communication or uterine rupture, the outcome might have been different.

In conclusion, this unique case of successful pregnancy following the management of NCRHP highlights the importance of early diagnosis and treatment of NCRHP for preventing severe complications and ensuring successful pregnancies using ART. Clinicians should maintain a high index of suspicion for NCRHP and utilize various diagnostic modalities for early identification.

## Data Availability

All data generated or analysed during this study are included in this published article.

## Abbreviations

NCRHP: Non-communicating rudimentary horn pregnancy
ART: Assisted reproductive technology
IVF: In vitro fertilization
b-hCG: Beta-human chorionic gonadotropin
TVS: Transvaginal sonography
MRI: Magnetic resonance imaging
IVP: Intravenous pyelogram
